# Qigong Exercises for the Management of Type 2 Diabetes Mellitus

**DOI:** 10.3390/medicines4030059

**Published:** 2017-08-09

**Authors:** Amy L. Putiri, Jacqueline R. Close, Harold Ryan Lilly, Nathalie Guillaume, Guan-Cheng Sun

**Affiliations:** 1 Institute of Qigong and Integrative Medicine (IQ & IM), 10127 Main Place, Suite B, Bothell, WA 98011, USA; amy.putiri@gmail.com (A.L.P.); Jackie@SeattleHealingAcupuncture.com (J.R.C.); haroldryanlilly@gmail.com (H.R.L.); nathalieguillaume@me.com (N.G.); 2 Seattle Healing Acupuncture, LLC, 1307 N 45th Street, Suite 204, Seattle, WA 98103, USA; 3 Healing Happy Hour, 930 Grand Concourse, Ground FL, New York, NY 10451, USA; 4 Bastyr University Research Institute, 14500 Juanita Dr. NE, Kenmore, WA 98028, USA

**Keywords:** medical qigong, type 2 diabetes mellitus, xiao ke, acupuncture and oriental medicine, Traditional Chinese Medicine (TCM), integrative medicine, Yi Ren Qigong

## Abstract

**Background:** The purpose of this article is to clarify and define medical qigong and to identify an appropriate study design and methodology for a large-scale study looking at the effects of qigong in patients with type 2 diabetes mellitus (T2DM), specifically subject enrollment criteria, selection of the control group and study duration. **Methods:** A comprehensive literature review of English databases was used to locate articles from 1980–May 2017 involving qigong and T2DM. Control groups, subject criteria and the results of major diabetic markers were reviewed and compared within each study. Definitions of qigong and its differentiation from physical exercise were also considered. **Results:** After a thorough review, it was found that qigong shows positive effects on T2DM; however, there were inconsistencies in control groups, research subjects and diabetic markers analyzed. It was also discovered that there is a large variation in styles and definitions of qigong. **Conclusions:** Qigong exercise has shown promising results in clinical experience and in randomized, controlled pilot studies for affecting aspects of T2DM including blood glucose, triglycerides, total cholesterol, weight, BMI and insulin resistance. Due to the inconsistencies in study design and methods and the lack of large-scale studies, further well-designed randomized control trials (RCT) are needed to evaluate the ‘vital energy’ or qi aspect of internal medical qigong in people who have been diagnosed with T2DM.

## 1. Introduction

Type 2 diabetes mellitus (T2DM) is a complex, chronic metabolic disease with hyperglycemia arising from insulin resistance, progressive pancreatic beta-cell failure and insufficient insulin secretion. T2DM is often associated with hypertension, dyslipidemia and atherosclerosis, and if not managed, patients may experience complications including stroke, cardiovascular disease, peripheral vascular disease, neuropathy and kidney failure. According to the International Diabetes Federation (IDF), more than 415 million people worldwide have diabetes, and over 642 million people are projected to have diabetes by 2040 [1]. Diabetes causes more deaths a year in the United States than AIDS and breast cancer combined [2,3]. T2DM can substantially diminish quality of life, decrease life expectancy and increase health problems and healthcare costs. The estimated total economic cost of diagnosed diabetes in 2012 in the United States was $245 billion. This estimate highlights the substantial burden that diabetes imposes on society [4,5]. The incidence of T2DM continues to rise by epidemic proportions and has emerged as a global health crisis. 

There is a growing body of literature suggesting a role for epigenetic factors in the complex interplay between genes and the environment, particularly in common disorders, like T2DM. Recent studies have shown lifestyle interventions, including diet and physical exercise, to be significantly more effective than metformin in reducing the incidence of T2DM [6,7,8,9,10]. Personalized self-care practices, such as qigong, may enhance the benefits of lifestyle modifications. Medical qigong, a specific form of qigong (Section 2), may have the potential to alter the epigenome and to prevent or reverse T2DM-related epigenetic abnormalities, thereby promoting healing and the quality of people’s lives. 

A thorough literature review searched indexes from 1980–May 2017 using the MeSH and Boolean search phrases of “Qigong”, “Qi-gong”, “Chi gung”, “Chi gong” AND “Diabetes”, “Type 2 Diabetes”, “Type 2 Diabetes Mellitus”, “Diabetic”. The databases search included PubMed, MEDLINE, Cochrane, NIH, EBSCO, CINAHL, clinicaltrials.gov, Google Scholar, the Qigong and Energy Medicine Database (Qigong Institute), OVID and the Wiley Online Library. In addition, articles were located by hand searching review and meta-analysis articles (Tables 2 and 3). All review articles located in English were used in this report. 

Previous studies suggest that medical qigong may be a beneficial adjunct treatment for individuals with type 2 diabetes mellitus (T2DM) and have shown consistent and statistically-significant positive associations between participation in qigong and blood glucose, triglycerides and total cholesterol [11]. Qigong has also been associated with trends in weight loss, reduced BMI and improved insulin resistance in people with T2DM [12]. Systematic reviews have found support for a role of qigong in the management of T2DM [11,13,14,15]. This retrospective literature review article aims to define medical qigong and its role in treating and healing patients with T2DM and to identify appropriate control groups and criteria of subjects to perform adequate randomized controlled trials in this field in an effort to eliminate this global public health epidemic.

## 2. Medical Qigong Terminology

Qigong (pronounced “chee gung”) is a branch of Traditional Chinese Medicine (TCM) and can be considered as an ancient practice of mind-body integration and refinement of one’s vital energy or life force for optimal health and for personal development [16]. The practice of qigong combines breathing, movement and meditation and therefore is often classified by Western providers under the category of “mind-body medicine”. Mindfulness is incorporated in the beginning training of qigong as practitioners develop awareness. Some contemporaries consider qigong as “meditative movement” [17] or “movement-based embodied contemporary practice” [18]. The unique practice of qigong is the oneness of mind-body through authentic qi (vital energy or life force) cultivation. This maturation of a peaceful mind permits the practitioner to connect with their authentic emotions by deleting old, negative thought patterns, as well as letting go of all unwanted feelings that may interfere with their experiences. When a person practices qigong, she or he can experience the vital energy or life force alive as a sense of resistance between their hands, electrical current flow or warm water flow in their internal body. 

Medical qigong is defined as the system of authentic qi (vital energy) practice, which empowers the body to heal itself and to facilitate the healing process of others. It involves appropriate management and regulation of all energetic and informational communications and interactions within and without the body in the process of self-healing and healing others. 

Medical qigong consists of two aspects of authentic qi practice, namely internal medical qigong and external medical qigong. Internal medical qigong refers to self-healing and self-care qigong practice or qigong exercises practiced by oneself. Internal medical qigong exercises include unique breathing methods, movements for specific health conditions, meditations with unique mudras and internal observations with specific energetic codes and images for healing health conditions. The movements, hand positions and breathing techniques are all done with intention and with the sensation and awareness of authentic qi. External medical qigong refers to a medical qigong practitioner facilitating the healing process of others. In external medical qigong, the practitioner works with his or her client by cleansing and clearing unhealthy qi, removing blockages or qi stagnation from the client’s energy field to promote healthy internal qi circulation, or by projecting specific healing qi into the energy field of the client and directing it to specific areas and systems of the body for restoring internal balance and harmony. The medical qigong practitioner may also transmit universal healing energy and energetic intelligence to their client for improving health conditions and restoring well-being. 

### 2.1. The History and Development of Medical Qigong

The term “medical qigong” was popularized by Guizhen Liu in the 1950s and became part of Chinese mainstream culture. Mr. Liu was famous for teaching nieyang-gong (inner-nourishing qigong), and at that time, medical qigong became the standard term for healing and improving health conditions with authentic qi cultivation, regulation and management. 

According to historical documents, qigong has been practiced and studied for about five thousand years in China. The following statement was recorded in the Yellow Emperor’s Classic of Internal Medicine, Suwen Chapter One: “If your mind is calm and peaceful and able to reach a state of emptiness, and you hold your spirit within, and your authentic qi flows easily and freely, how could illness arise?” (the reign of the Yellow Emperor was 2690–2590 B.C.) [19]. Jing Zuo literally means “quiet sitting” or “sitting in silence” and refers to a meditation practice to achieve a peaceful and tranquil mind for internal observation, cultivation and realization. An important part of the Yellow Emperor’s Classic of Internal Medicine is the Ling Shu (Spiritual Pivot), which contains detailed information about the acupuncture meridians. The acupuncture meridians were discovered by qigong practitioners in ancient times and can be identified with internal visual and experiential perception. 

Lao Zi (601–531 B.C.), the founder or “father” of Daoism, mentioned authentic qi cultivation in his classic Dao De Jing (Classic on the Dao of Virtues). He emphasized that the way to obtain health was to “concentrate on authentic qi cultivation and become more flexible.” The famous philosopher Zhuang Zi, in his book Nan Hua Jing (3rd Century B.C.), stated that “the immortals’ breathing reaches down to their heels and the normal person’s breathing to the throat.” He emphasized the importance of breathing in the internal cultivation. To this day, one of the commonly-accepted definitions of qigong is breathing exercise. Xing Qi refers to “moving the qi”. An early description of this type of qigong practice is called “The Jade Pendant Inscription on Xing Qi” (6th Century B.C.). 

The Jade Pendant Inscription on Xing Qi could be the earliest monograph on qigong in Chinese history. It reads as follows: “In moving qi, one breathes deeply and cultivates the authentic qi within. If the authentic qi is stored, it expands. When the authentic qi expands, it descends down to the Dantian (energy center in lower abdomen). When the authentic qi becomes stable in the Dantian, it will solidify. When the authentic qi is solidified it will begin to sprout and move to the root of the Du meridian. After the authentic qi has moved to the root of the Du meridian it will grow and rise up along the Du meridian. When it rises up to the top of the head, it will flow down along the Ren Meridian to the bottom of the torso. When the authentic qi rises up to the top of the head, it reaches heaven. Heavenly qi functions from above and earthly qi functions from below. Moving the authentic qi around the Du and Ren meridians freely lead to vitality and longevity while adverseness to this leads to aging and death.” 

Medical Qigong has been practiced with documented results in China. For example, 156 different qigong therapy methods for healing specific illnesses were described in an ancient book, entitled “Treatise on the Causes and Manifestations of Diseases” (610 A.D.) by Yuan-Fang Chao. In this book, Dr. Chao summarized 1729 cases of diseases from clinical experiences and pathologic observations, and explained the symptoms of diseases and their causes as they relate to internal subtle energy flow. For treating diseases, the unique method of Yuan-Fang Chao was to give patients specific qigong exercises for their self-healing and self-care. 

It was during the Jin dynasty (A.D. 265–420) and the Northern and Southern dynasties (A.D. 420–589) that qigong developed as a practice to prevent or correct disease through the transmission of healing qi [19]. Medical qigong therapist can emit their healing qi to assist another person. This transmitted qi has healing intelligence that can communicate with another person’s qi for the purpose of addressing specific health conditions [20].

### 2.2. Definitions of Internal Medical Qigong

There are thousands of types of internal qigong, and they can vary in focus from marital arts, sports, health, spiritual growth and more. They also vary in movements, intention, duration, frequency, intensity, instruction, technique, etc. With such a wide variation of qigong available, it can be difficult to understand what qigong truly is, how it is different than physical exercise and what effect it can have on the diabetic condition. 

The four available systemic reviews on the effectiveness of qigong for diabetes all concluded that there was large variation in qigong exercises and poor definitions and descriptions about the specific type of qigong used (Table 1) [11,13,14,15].

You can directly see many of these definitions and differences in the individual studies outlined in Table 2 and Table 3. Many, if not all of these studies define qigong using a combination of breathing, movement, stretching and form. If this is the full definition of qigong, it must be asked if qigong is any different than physical exercise. Is it not true that physical exercise also emphasizes breathing, movement, stretching and form?

### 2.3. Yi Ren Medical Qigong 

The most current definition of Yi Ren Medical Qigong (YRMQ) is “authentic qi production, management and regulation”. When we look further, the qigong definition and form defined by Sun contains something much different that separates its qigong study from the others and that of physical exercise and breathing techniques. Sun explains that qigong is an energy medicine practice based on the “cultivation of authentic qi or vital energy in the body” [21]. It does not just work on the physical body through muscle movements, tendon stretching and breath control to manage blood sugar levels. It works on the qi/vital energy and the channels and organs within the Chinese medicine system that influence the function of the organs involved in the diabetic condition including the pancreas, liver, kidneys and autonomic nervous system [21]. YRMQ can be considered as both static and dynamic. In the beginning of training, the exercise and energetic movements are more dynamic, and once the system is activated, the exercises become more static, while the energy remains dynamic.

### 2.4. Yi Ren Medical Qigong Exercises for Healing T2DM

In the Yi Ren Medical Qigong Manual for Healing Type 2 Diabetes Mellitus, specific organ exercises are outlined step-by-step with detailed movements and hand positions/mudras that correlate with each meridian and internal organ affected in T2DM. The manual also contains specific exercises and information on how to increase one’s amount and awareness of qi/vital energy, which will be used throughout each T2DM specific exercise. Each session opens with 1–2 min of gentle shaking or bouncing, which helps release energy in the yin and yang meridians in the front and back of the body, respectively, in addition to the internal organs. The next set of exercises focus on activating authentic qi through the Turning on the Internal Power Station Exercise and the Energy Field Constructive Exercise [21].

Once the energy is activated, which has been clinically demonstrated among Yi Ren Qigong students and practitioners, they are able to move into exercises specifically designed to help balance their energetic system. These qigong exercises include (1) Parasympathetic Nervous System Empowering Exercise; (2) Excess Liver Energy Releasing Exercise; (3) Pancreas Empowering Exercise; (4) Liver and Pancreas Harmonizing Exercise; and (5) Internal Power Station (Kidney) Activating Exercise.

Why is qi or vital energy so important and unique and how will this benefit a diabetic person? It is because qi, especially within the body, is more than the ability to do work and to carry out functions such as insulin production, release and absorption. Qi contains important information such as memories and emotional responses, whether good or bad, that lead to different lifestyles, habits and ultimately to different gene expression. For example, pancreas qi not only helps the pancreas to function properly and produce insulin appropriately, but also contains information about dietary habits and preferences along with one’s emotional response to eating. Thus, if we can change or improve the pancreas qi through specific qigong exercises, we see not only an improvement in insulin production, but also in positive dietary and behavior changes, both of which are necessary to maintain or improve T2DM. From this prospective, qigong can be considered as a behavior-modification tool to assist patients in changing and transforming unhealthy habits into healthy ones.

## 3. Traditional Chinese Medicine, Medical Qigong Etiology and Pathophysiology

Traditional Chinese Medicine (TCM) and medical qigong view the body as a whole and recognize the importance of the interrelationship between the internal organs. Communication between different parts of the body is not only maintained through the circulatory and nervous systems, but also though energy pathways called meridians. These meridians create an internal energy network connecting the organs, tissues and cells and can also be referred to as the bio-energetic-information system. 

From the medical qigong perspective, the organs and systems associated with the development of T2DM are the liver, pancreas, kidneys and the autonomic nervous system [21]. To fully understand the pathophysiology of T2DM from this perspective it is important to first acknowledge the functions of these internal organs and systems and how they influence each other through the meridian/bio-energetic-information system. It is also important to note that, along with the physical functions of the internal organs, TCM recognizes that thoughts and emotions are connected and affect the function of each organ system [19]. For example, if one experiences excess amounts of anger, their liver system is negatively affected, grief and sadness challenges the lung function, fear and hatred deplete the kidneys and worry, overthinking, shame and guilt all have adverse effects on the pancreas [21,22,23].

### 3.1. Pancreas

The pancreas functions to transform and transport nutrients [22,23]. The pancreas, as understood from the Western medical perspective, functions to break down or transform food into products our body can use as energy. The energetics of the pancreas work similarly to the physical nature of the organ, though the energy integrates on a subtler level. The nutrients produced from this process are then transported to the lungs where they mix with oxygen and become a usable form of qi or vital energy, which can be distributed to the internal organs and tissues of the body [21,22,23]. Please note that in traditional texts, the spleen was considered as the organ that worked with the stomach for digestion; however, from the perspective and experience of YRMQ practitioners, the pancreas is responsible for digestive function and is intrinsically involved with the development of T2DM. In this article, we will use the pancreas meridian. 

If the pancreas is not functioning at an optimal level, as with most T2DM patients, the transformation and transportation of nutrients and fluids will also be limited, resulting in what TCM calls dampness. Dampness is a byproduct of a pancreas deficiency with symptoms such as foggy thinking, abdominal bloating, cloudy urination, weight gain especially around the abdomen and excess thinking and worry [22,23]. It is brought on by overeating, consuming cold and raw foods, consuming carbohydrates and sugars in excess and lack of exercise. One of the causes of dampness from a biomedical viewpoint is an accumulation of too much glucose in the blood [24].

The emotions and habits that affect the pancreas are: worry, overthinking, shame, guilt, self-blame, self-punishment and lack of professional and personal boundaries [21]. Overactive emotions or emotional blocks may disrupt the energetic balance of the organ and, over years of stress, will present physically with a lack of insulin production. 

The root cause of T2DM is the pancreas and stomach qi deficiency. When the pancreas and stomach are not functioning properly due to deficiency, the transformation and transportation of food and drink are inhibited. This pattern is common in people who are pre-diabetic and have a difficult time regulating blood sugar levels. Symptoms include fatigue, muscle weakness, craving sweet foods, abdominal distension, spontaneous sweating, difficultly regulating blood glucose levels, frequent urination and possible glucosuria [24]. In more advanced cases, additional symptoms will be present such as dry mouth and thirst with no desire to drink [25].

Medications such as metformin are beneficial at this stage of T2DM. Metformin helps to regulate blood glucose levels by increasing insulin sensitivity and lowering glucose production in the liver [26,27]. However, from a TCM point of view, metformin has the energetic properties of cold and bitter, and the pancreas can be depleted by these properties, thus impairing its overall function. The effects of the drug will lessen, over time, as it depletes the energy of the pancreas [24].

### 3.2. Liver

The liver is in charge of storing the blood and maintaining a free flow of qi (vital energy) throughout all of the organs. Liver qi can also affect the pancreas and digestion. When the liver energy is stagnant due to excess emotional stress, improper dietary habits such as too much alcohol consumption or lack of exercise, it directly affects the function of the pancreas. Liver qi stagnation is a common pattern in T2DM since there is a build-up of fat, or in TCM terms dampness, in the liver. The liver (wood element) becomes hyperactive or excessive, and due to the hyperactivity, it then releases too much energy into the pancreas and stomach (earth element), causing the pancreas to become deficient and/or an accumulation of heat in the stomach. Symptoms include: nausea, abdominal distention, reflux, vomiting or diarrhea [23]. The communication and balance of energy between the liver and the pancreas is extremely important in the overall health of the individual and plays a major role in T2DM.

### 3.3. Liver and Pancreas Disharmony

Liver and pancreas disharmony is a common pattern linked to stress and seen in the earlier stages of T2DM. The five-element controlling cycle represents this pattern and describes it as the liver (wood element) overacting on stomach and pancreas (earth element). The liver becomes stagnant with higher levels of stress, irritability and anger and “overacts” or impairs the function of the stomach and pancreas [23]. Stagnation will generate heat leading to symptoms of irritability, red or dry eyes, thirst, dry throat and constipation [24,25]. Other symptoms of liver and pancreas disharmony are abdominal distention, alternating constipation and diarrhea, flatulence, heartburn and elevated blood glucose levels [24].

A proper diet will support the pancreas; however, the root issue is stagnant liver qi, creating a deficient pancreas. This pattern directly correlates with stress; therefore, exercise is a more appropriate form of treatment, more so than diet. It is recommended to eat a healthy diet, but aerobic exercise 1–2 h/day will be the most beneficial to move liver Qi stagnation and to clear heat [24].

### 3.4. The Kidneys

The kidneys are said to be the foundation of yin and yang for all other organs. Kidney yin energy and kidney yang energy work together to support the internal organ system. Kidney yin represents the fluids within the kidneys, and kidney yang is the energy that provides the action of the transportation and transformation of fluids. In TCM, it is understood that as the kidneys and lifegate (a vital energy center) decline, all of the internal organs will be affected [23]. A deficiency of kidney energy may lead to an increase in insulin resistance in people with T2DM. 

### 3.5. Pancreas Qi and Kidney Yin Deficiency

The pancreas qi and kidney yin deficiency pattern is indicative of chronic stages of T2DM. Most people who are diagnosed with T2DM will have pancreas qi and kidney yin deficiency. Patients will be taking one or more hypoglycemic agents, such as metformin, and in more severe cases, occasional or consistent insulin [24].

### 3.6. The Autonomic Nervous System

The autonomic nervous system (ANS) is comprised of two extraordinary meridians, the reservoirs of energy for the internal organ meridians: the conception vessel (Ren meridian) and the governing vessel (Du meridian). The Ren meridian is considered the “sea of yin”, as it links all of the yin meridians and regulates the yin energy of the body. The Du meridian is referred to as the “sea of yang” as it links all of the yang meridians and regulates the yang energy of the body [28]. Yi Ren medical qigong associates the Du meridian with the ANS, the brain, kidneys and reproductive system [29]. It associates the Ren meridian with the parasympathetic nervous system (PNS), often referred to as the “rest and digest” part of the ANS, mainly regulating the yin energy of liver, kidneys and pancreas [29]. A lack of relaxation and over stimulation, due to the modern day lifestyle such as excess use of electronics, stress, trauma, etc., create an environment that will keep the sympathetic nervous system (SNS) activated, causing a “flight or fight” response in the body. This overstimulation of the SNS will also suppress the restorative functions of the PNS [30].

Both T2DM and obesity have been linked to an overactive SNS [31]. Dysregulation of the ANS may be associated with insulin resistance [32]. Therefore, a balanced ANS is a key factor for the treatment of T2DM as it supports the proper functioning of the organs involved in the disease. Exercise, qigong, yoga, massage, mindfulness training and meditation are forms of relaxation techniques that may be implemented for stress reduction.

## 4. Effects of Medical Qigong in Adults with Type 2 Diabetes Mellitus

A randomized controlled pilot study was conducted at Bastyr University in 2008–2009 implementing a diabetes-specific protocol based on the previously-mentioned energetic assessment of the typical diabetic patient. In this uniquely-designed, three-armed, 12-week randomized, controlled clinical trial, researchers investigated the effects of qigong on glucose control in type 2 diabetes and compared the biological and psychological responses of YRMQ with progressive resistance training (PRT) or standard care in patients with T2DM. [12,32,33]. The results on blood glucose control [32], perceived stress [33], body weight and insulin resistance [12] in people with T2DM were interesting. Fasting plasma glucose, body weight, body mass index (BMI), insulin and perceived stress levels were recorded before and after the 12-week intervention. As previously reported, plasma glucose levels for individuals indicated significant reductions in the qigong group. In contrast, the plasma glucose levels showed an increase in both the PRT group and the control group, respectively [32].

The homeostatic model assessment for insulin resistance (HOMA-IR) is an indirect calculation of insulin resistance (IR) using insulin and glucose levels. HOMA-IR shows reasonable correlations with the gold standard, hyperinsulinemic euglycemic clamp technique [34]. As previously reported, PRT group participants demonstrated significant body weight loss and BMI decrease, but their insulin resistance increased; in contrast, the YRMQ group showed a smaller loss in body weight and BMI, but their insulin resistance decreased [12]. The results of the preliminary study showed a very interesting phenomenon suggesting that medical qigong works with a different mechanism for the management of type 2 diabetes compared with PRT.

### 4.1. Possible Mechanisms of Action

Insulin resistance plays a major role in the pathogenesis of metabolic syndrome and T2DM, and yet, the mechanisms responsible for it remain poorly understood. Current studies have shown that mitochondria may be a major factor in the development of T2DM [35,36,37,38]. Mitochondria are known as the powerhouses of the cell as they convert energy from the food we eat into an energy substance called adenosine triphosphate (ATP) through the process of oxidative respiration. A potential mechanism linking mitochondrial dysfunction to insulin resistance is seen when decreases in substrate oxidation affect electron flow through the electron transport chain (ETC) and lead to the formation of superoxide, which damages mitochondria [37]. Mitochondrial superoxide shows evidence of being a cellular reaction to nutrient excess, leading to insulin resistance with body tissues [38]. Exercise is also known to have a major impact on both mitochondrial function and insulin sensitivity in skeletal muscle. Exercise increases energy expenditure and insulin sensitivity. Studies suggest that aerobic exercise may be useful in reversing insulin resistance in pre-diabetic individuals, possibly preventing the development of type 2 diabetes [39].

Muscle insulin sensitivity has been studied extensively with conflicting results [40]; however, the mechanism of action is thought to occur in the glucose transport across the cell membrane of skeletal muscles via the glucose transport proteins, GLUT1 and GLUT4 [41]. GLUT1 supports the basal glucose transport, while GLUT4 increases glucose transport in response to insulin and contraction. In 2001, Gaster et al. identified a decrease in GLUT4 density in insulin-sensitivity in skeletal muscles of T2DM patients [42]. Furthermore, studies investigating the role of exercise in individuals with T2DM often relate the benefit of reduced blood glucose following the increase in GLUT4 through skeletal muscle contraction. In the Xin et al. review, the authors discuss the possible mechanism of action of the qigong exercises as a combination of muscle movement and stress reduction properties [11]; however, in the YRMQ study, participants in the PRT group did not demonstrate reduced levels of glucose or insulin levels. This may be a result of the low intensity of the PRT exercises. It has been demonstrated that the more intense the exercise, the better the results; however, individuals who are diagnosed with T2DM are typically obese; thus, intense exercise is not feasible in this population [40].

One possible explanation for the outcome in the YRMQ study [12] for the YRMQ participants who experienced a reduction in insulin resistance is that in medical qigong practice, specific qi-energy breathing exercises that are uniquely recharging might enhance the mitochondrial oxidative respiration process and provide energetic support to mitochondria for preventing ETC electron leakage, thereby restoring the functions of the mitochondria for reversing insulin resistance. The possible mechanism between medical qigong practice and supporting the function of mitochondria and decreasing insulin resistance needs further study. 

In addition to insulin resistance, it is widely accepted that the etiology of T2DM involves pancreatic beta cell dysfunction and insulin resistance. In molecular genetics studies, the most common mutation leading to mitochondrial diabetes is the A3243G mutation in the mitochondrial encoded tRNA. Researchers have found that the A3243G form of mitochondrial diabetes is related to decreased glucose-induced insulin release, but is not characterized by insulin resistance, indicating that the major pathology occurs within mitochondria of pancreatic beta cells [43,44,45]. In the YRMQ study, the YRMQ group participants practiced, among other qigong exercises, a specific medical qigong exercise for empowering the pancreas, which might be beneficial for restoring the functions of the mitochondria of pancreatic beta cells. Further studies are needed to investigate these possible benefits.

## 5. Designing Adequate Randomized Controlled Clinical Trials

### 5.1. Description of Qigong Exercises, Frequency and Duration

Details regarding the type of training utilized in clinical trials is necessary for replicability and comparability. Qigong style, duration of study, study results, attributes and limitations are described in Table 2 (RCTs only) and Table 3 (other trial forms). In addition to limitations that already exist is certain study designs, qigong studies pose unique challenges, including the accessibility to measure and see the qi/vital energy and the inability to blind subjects.

Descriptions should include whether the style is static or dynamic, the duration of study, frequency of practice, predominant movements and adherence to protocol [13]. Chen et al. included many studies of varied duration from 1 month to 6 months and provide little information regarding the subjects. Full articles of studies indicated in this review and in the Lee et al. review were difficult to find in English texts. Chen et al. (Table 1) also included both qigong and Tai Chi studies; however, the styles are indicated along with the duration and frequency [14]. This is the first publication that includes the YRMQ exercise descriptions. Among the studies indicated in Table 2, the frequency of exercises range from three-times per week to daily from 1–2 h. Some of the more recent studies [48,49,50] included the greatest amount of detail regarding the practice sessions, which includes a brief description of the exercise and the length of time devoted to each exercise or section, and information regarding intensity is also helpful. 

Of the six RCTs listed in Table 2, five styles of qigong and five countries are represented, including Eight-Section Brocade/Japan [47] also known as Ba duan jin/China [50], KaiMai/Australia [48], Yi Ren Medical Qigong/USA [32], qigong relaxing/Japan [46] and Tai Chi Qigong/Southern Thailand [49]. The uncontrolled observational studies indicated in Table 3 include Five Element Gymnastics/Taiwan [53], qigong walking/Japan [51] and KaiMai/Australia [52]. The frequency and duration of the qigong exercises varied between studies. Iwao et al.’s study design of three days was the shortest duration [51], while the Xiao et al. study consisted of the longest duration of 24 weeks [50]. The qigong practice sessions ranged from 20 min per day to 1–1.5 h daily. All studies reported benefits to practicing qigong on blood glucose management with varying degrees of improvement. 

With regard to study duration, a three-month length of study is imperative to generate results from the individual’s bloodwork for HbA1c levels. Surprisingly, many studies, including the Iwao study, were less than three months in duration and demonstrated improvement in glucose management [51]. Frequency of practice varied in all of the studies identified in systematic reviews; however, all showed improvement in glycemic control. In a fairly recent study looking at the effects of qigong in patients with fibromyalgia, researchers collected information regarding the individual’s practice and adherence to the study protocol and found that those who followed the protocol of 5 h per week showed a significantly greater improvement in pain and other measures when compared with those who engaged in minimal practice [54]. Additionally, researchers of a chronic fatigue syndrome study also demonstrated a direct relationship between the amount of qigong practiced and symptom improvement [55]. It is anticipated that T2DM patients would also see more improvement in symptoms with increased frequency of practice. Differences in degree of improvement may also exist between practice settings: group or instructor-led versus home-based or individual practice. Most qigong studies implemented a combination of the two approaches with promising results; however, in a 2005 exercise study, Dunstan et al. concluded that supervised training followed by home-based training was insufficient to produce sustainable benefits in glycemic control [56]. Future research is needed consisting of detailed data collecting regarding the exercise setting, time, length and duration for each participant not only to confirm protocol compliance, but also to determine if correlations exist between the variables.

### 5.2. Criteria for Enrollment

In order to perform a robust and generalizable RCT evaluating the effects of qigong in adults with type 2 diabetes, the following criteria for enrollment of subjects is suggested: (1) diagnosed type 2 diabetes according to the most current standards (ADA 2016) of HbA1c ≥6.5% [57]; (2) non-insulin dependent; (3) free of diabetic complications; (4) adults ≥18 years old; (5) combination of male and female participants; and (6) maintaining the same medication, diet and exercise use through the duration of the study. It is important that authors include this information in their reporting of data in order to allow for study replication and comparison. 

Part of the development in investigating new or, in this case, ancient interventions to warrant larger RCTs is initially based on the analysis of quality case reports. Case reports on the effects of qigong on T2DM show insufficient reporting of the subjects and limit researchers in their ability to define the subjects at hand. Many of these case reports have been summarized in an analytical review published in 2009, and many beneficial results were demonstrated [14]. Many earlier case reports presented at various conferences tended to lack other information regarding their research subjects and their criteria for enrollment. 

Apart from the obvious lack of a control group in the studies indicated in Table 3, these studies tended to be void of other pertinent information required to replicate a study, such as little information known about the subjects, study of short duration (less than three months) and information lacking regarding participant use of medications, diet or additional exercise. In turn, many authors of review articles state that studies to-date in this field are generally of low methodological quality [11,13,14,15].

The most important criteria that must be met in order to properly ascertain the effect of qigong in patients with type 2 diabetes is the diagnosis of type 2 diabetes. Many studies involved adults with elevated blood glucose levels [48] or pre-diabetes and not necessarily patients diagnosed with T2DM. In a review article, reviewers included studies of patients with type 1 diabetes [11]. 

Only two studies stated that subjects must keep oral medications, diet and exercise regimes the same for the duration of the study [32,48]. In the Tsujiuchi study, the authors note that diet and exercise were not modified in the study; however, no mention was made regarding the monitoring of these confounders [46]. Changes in medications may be recommended by a patient’s primary care physician if changes in bloodwork are seen. Though metformin was documented to not attenuate the effects of exercise [58], with the understanding of the energetic qualities of metformin, it may be affected by medical qigong practice. It is hypothesized that the enhanced energy flow in the body may actually enable medications in the body to work better or, in some cases, the body may be more sensitive to the medication.

The well-designed Youngwanichsetha study [49] provided adequate information regarding their subjects, but their study is limited in generalizability as a result of observing the qigong effects in only postpartum women, who were much younger in age, and the duration of disease was much less than the rest of the RCTs published to-date [49]. Though no differences in baseline characteristics were found between groups, researchers also included those with complications. In turn, it is difficult to compare this study with other qigong studies evaluating the effectiveness in type 2 diabetes [59]. 

### 5.3. Selection of Appropriate Control Groups

Randomized controlled trials (RCT) are considered the gold-standard for evaluating the effects of proposed interventions [60]. Identifying and implementing well-designed control groups for research studies increase the reliability and validity of the intervention analyzed. In addition, control groups improve the ability to blind subjects and researchers during a study [61]. For instance, as in pharmaceutical studies where placebos or sugar pills are often used as controls and in acupuncture studies, sham or fake acupuncture may be used as controls. Sham acupuncture is performed by inserting needles in the body similar to an acupuncture treatment, but are not defined acupuncture points or along internal energy meridians [62] or the needles may not be inserted fully. Since it is not always appropriate or feasible to blind subjects with qigong studies, conventional exercise may be considered as a control group. Combining conventional exercise forms with meditation or movement with mental instruction, as in “meditative movement” [17] or “movement-based embodied contemporary practice” [18], may also be considered to serve as adequate controls in order to differentiate the effect of qi versus exercise movements. Qigong without authentic qi is simply exercise. 

Earlier qigong studies were case reports and did not contain control groups. These studies were primarily conducted in China, and generally, only abstracts can be found easily for English speakers. All of the case reports demonstrated positive effects of qigong with adults with type 2 diabetes or pre-diabetes [14]. Unfortunately, uncontrolled studies make it difficult to evaluate the effectiveness when compared to patients following standard care, including diet, exercise and medication, from their medical doctor. Huang et al. describes their study as quasi-experimental, but they did incorporate a control group [53]. The three uncontrolled observational studies identified in this thorough search demonstrated a significant improvement in managing blood glucose levels [51,52,53].

Usual or standard care control groups are used in more recent qigong research studies [32,46,48,49,53,63]. All of the RCTs located, including the quasi-experimental study [53], exhibited a consistent benefits with qigong. A significant decrease in HbA1c levels was detected over a 12-week–24-week period of time. It must be noted that Fang et al. randomized subjects into two groups; however, the control group was exposed to the qigong exercises for two months, while the intervention group continued to practice qigong for an additional two months [47], which prevents investigators from making appropriate between-group comparisons. However, the results indicated a decrease in HbA1c levels from pre to post (four months). Further randomized controlled trials are needed to investigate qigong effects in this population in order to contribute to this field of evidenced-based science. 

In the YRMQ study, the researchers created not only a non-intervention control group, but also an active control group [32]. The low-intensity PRT exercises implemented mimic the movements of qigong without the qi (vital energy), the key to the beneficial results of YRMQ. In this study, the purpose of the active control group was two-fold. First, the active control group served as sham qigong, and second, it enabled researchers to replicate previous studies involving the investigation of progressive resistance training in individuals with T2DM. PRT has been studied with conflicting results; however, the more intense the exercise, the better the results, but more patients experienced adverse events or dropped out of the study [40]. The standard care control group, represented in the third arm of the study, provided a comparative group for the two exercise interventions. Surprisingly, no other qigong research studies to-date include an active control group to differentiate the actions of qigong versus regular exercise movement. 

## 6. Limitations of Review

Limitations to this prospective literature review may include the lack of systematic review, though systematic reviews to-date have been considered and limit the ability to detect bias. The use of only English-published articles limits the number of studies analyzed.

## 7. Conclusions

Though research in the field of qigong has improved tremendously over the past couple of decades and preliminary data are promising, longer, adequately controlled trials are warranted to evaluate the effects of medical qigong with patients diagnosed with T2DM. Once the effects of medical qigong have been isolated, the next step would be to design an integrative model that focuses on treating the whole person using a variety of modalities to individualize their treatment based on an energetic diagnosis. Qigong is a more feasible way to heal from the complications associated with T2DM. Modalities may include medical qigong, nutrition therapy, exercise, acupuncture, Chinese and Western herbs, pharmaceuticals and naturopathic medicine.

## Figures and Tables

**Table 1:** Summary of definitions for internal qigong in systematic reviews.

Systematic Review	Discussion on Internal Qigong Variation
Xin (2007) A qualitative review of the role of qigong in the management of diabetes [11]	The included studies were of different styles of static and/or dynamic qigong and varied in duration (from 10 days to more than three years) and frequency. “The quality of reporting of studies of the effect of qigong on diabetes management in the published literature requires improvement, including more explicit description of the type of training used (static versus dynamic, duration, frequency, pre-dominant movements, etc.), to allow replication and comparability of effects between studies.”
Chen (2009) An Analytical Review of the Chinese Literature on Qigong Therapy for Diabetes Mellitus [14]	“Qigong comes in many forms. Not all forms are equally effective in treating diabetes… Many investigations of qigong therapy for DM even do not specify what form of qigong was used or how it differs from others, making reviews and evaluations even more difficult.”
Lee (2009) Qigong for type 2 diabetes care: A systematic review Complementary Therapies in Medicine [13]	“There are significant differences between the numerous forms of qigong, which poses difficulties in establishing quality standards for this treatment.28 In future trials, clear description of the qigong intervention should be provided together with a description of the level of expertise of the instructors.”
Freire (2013) Therapeutic Chinese exercises (qigong) in the treatment of type 2 diabetes mellitus: A systematic review [15]	“It can be seen that all the studies that identified the types of exercises practiced by the patients performed different qigong sequences with their patients. Therefore, regarding qigong research, not only the efficiency of qigong exercises in the treatment of the diabetic patients in the study, but also the efficiency of these specific qigong exercises in the treatment of diabetic patients.”

**Table 2:** Summary of randomized controlled trials (RCTs) Investigating the effects of qigong in adults with type 2 diabetes.

Study (Year) Location *Review article cited*	Nature of Practice	Amount of Practice	Study Results, Attributes and Limitations
TSUJIUCHI et al. (2002) [46] Japan/Tokyo *Xin 2007* *Lee 2009* *Friere 2013*	Qi-Gong relaxing consists of "controlled synchronized breathing with slow body movements as an aerobic exercise and relaxation".	4 months Weekly 2 h Qi-Gong group sessions held by a Chinese Qi-Gong doctor; all requested to practice at home	Compared with the Control period of Group 2, Group 1 demonstrated significant improvement of HbA1c level (*p* < 0.01) *Combination of Chinese Qi-Gong doctor* *led group practice and at home individual practice*
FANG (2008) [47] Japan/Tokyo *Friere 2013*	Eight-Section Brocade practice is a movement of limbs combined with breathing and mind concentration.	4 months Practice 1 ×/day at home for about 1 h Both groups given 2-month practice period Intervention group continued for another 2 months	HbA1c pre = 7.7 ± 1.55 HbA1c post = 6.9 ± 1.49; (*p* < 0.05) *Unable to compare intervention to control group since control group was exposed to qigong for 2 months.* *Little known about medications, diet and additional exercise in subjects.* *Detailed information regarding the exercises at end of article.*
SUN et al. (2010) [32] USA/Seattle, WA *Friere 2013*	Yi Ren Medical Qigong (YRMQ) defines qigong as traditional Chinese energy medicine practice combining breathing, movement, and meditation. "The term 'qi' (or 'chi') meaning 'vital energy of the body' and 'gong' meaning the skill and achievement cultivated through regular and disciplined practice."	12 weeks Weekly Qigong or PRT group sessions for 60 min/wk with certified instructors and practiced at least twice a week at home for 30 min following a DVD	All qigong intervention participants showed reduction in FBG in contrast to the control and PRT groups; YRMQ group showed significant reductions in FBG, slight reduction in weight and a trend toward reversing insulin resistance. *Specific exercise descriptions lacking from publications making it difficult to replicate study. Combination of Certified Qigong Instructor led group practice and at home DVD led individual practice.*
LIU et al. (2011) [48] Australia/Queensland *Friere 2013*	KaiMai-style qi-gong: 28 min warm-up 30 min practice 6–28 min cool-down intensity varied; length of time increased through duration of study	12 weeks 3 group sessions/week led by qigong expert Participants received DVD to practice at home 1–1.5 h/day on days group sessions did not meet	Between-group differences in favor of intervention in Weight (*p* < 0.01); Effect of qigong on improved IR was mediated by reduced weight *Combination of qigong expert led group practice and at ome DVD led individual practice.* *Qigong practice fluctuated throughout the study.*
YOUNGWANICHSETHA et al. (2013) [49] Southern Thailand	Tai Chi Qigong Group protocol: 1. warm-up with 25 movements 2. tai chi qigong following set one of Lin Housheng’s style comprising 18 movements for 30 min 3. cooling-down with 5 qigong movements for 5 min	12 weeks Intervention Group: 50 min tai chi qigong exercise program 3 ×/wk for 12 weeks plus 5 ×/wk at home –6 months postpartum Control Group: usual care	Statistically significant reduction in FPG, HbA1c and BP in intervention when compared with the Control Group (*p* < 0.05). No significant between group differences in body-weight or BMI at 12 weeks. *Subjects all post-partum younger women making study not generalizable, but helpful for this population. Combination of group and individual practice.*
XIAO (2017) [50] China/Bejing	Ba duan jin Qigong (BDJ) – 60 min training 10 min warming-up BDJ exercise 45 min 5 min cool-down	24 weeks (5) 1–2 h sessions/week	HbA1c (*p* = 0.034) at baseline and week 24 *Little known about medications, diet and additional exercise in subjects.* *Published photos to demonstrate qigong* *exercises along with step-by-step procedures.*

**Table 3:** Summary of other studies evaluating the effects of qigong in adults with type 2 diabetes.

Study (Year) Location *Review article cited*	Nature of Practice	Amount of Practice	Study Results, Attributes and Limitations
IWAO et al. (1999) [51] Japan/Kyoto *uncontrolled observational study*	Qigong walking = mild and slow walking using all muscles in body Conventional walking = normal walking	Evaluated 90 min post lunch on 3 different days Qigong walking (30–40 min) Conventional walking (30 min)	Plasma glucose decreased in both exercise groups Conventional = 228 to 205 mg/dL; Qigong = 223 to 216 mg/dL Compared with no exercise, 229 mg/dL (*p* < 0.025) *Very short-duration study where subjects served as their own controls. Little information known about subjects.*
LIU et al. (2010) [52] Australia/Queensland *Pre-post feasibility trial uncontrolled observational study*	Kai/Mai Tai Chi/Qigong style - defines qigong as "mind-body movement therapy…most involve combined training of movement, breathing, and mind, with a strong focus on the mind“	12 weeks 1–1.5 h 3×/wk with Tai Chi/Qigong instructor; also encouraged to practice at home	Good adherence and high acceptability; Significant reductions with BMI, HbA1c and IR *No subjects were taking medications. Combination of group and individual practice.*
HUANG et al. (2012) [53] Taiwan/Kaohsiung *quasi-experimental*	Defines qigong as breathing training. Qi meaning breath and gong meaning to train. Five Element Gymnastics (FEG) consolidates Qigong, Xiang Gong & Martial Arts with Gymnastics.	16 weeks FEG practiced following DVD 2 ×/day for 20 min each session for 16 weeks with 2 sessions in first week taught by certified fitness instructor and group education sessions at weeks 3, 7, 11 and 14; Control group maintained usual activities	HbA1C change -6.77 (*p* < 0.01) *Included insulin-dependent subjects; however, medications remained same. Combination of instructor led group practice and DVD led at home individual practice.*

## Supplementary Materials

Supplementary File 1
