# Supplementary material for: Qigong Exercises for the Management of Type 2 Diabetes Mellitus
Source: Medicines (Basel). 2017 Aug 9;4(3):59. doi: 10.3390/medicines4030059 (PMC5622394; doi:10.3390/medicines4030059)
Supplement: Supplementary File 1 [file medicines-04-00059-s001.pdf]

STUDY (YEAR)<br>LOCATION<br>*Review article cited*	NATURE OF PRACTICE	AMOUNT OF PRACTICE	STUDY RESULTS, ATTRIBUTES AND LIMITATIONS
**FANG (2008)**<br>**JAPAN/TOKYO**<br>*Friere 2013*	Eight-Section Brocade practice is a movement of limbs combined with breathing and mind concentration.	4 months<br>Practice 1x/day at home for about 1hr<br>Both groups given 2-month practice period<br>Intervention group continued for another 2 months	Analyzed (n=20)<br>HbA1c pre = 7.7+/-1.55 HbA1c post = 6.9+/-1.49;<br>(p<0.05)<br>*Unable to compare intervention to control group since control group was exposed to qigong for 2 months.*<br>*Little known about medications, diet and additional exercise in subjects.*<br>*Detailed information regarding the exercises at end of article.*
**LIU et al. (2011)**<br>**AUSTRALIA/QUEENSLAND**<br>*Friere 2013*	KaiMai-style qi-gong:<br>28min warm-up<br>30min practice<br>6-28min cool-down<br>intensity varied; length of time increased through duration of study	12 weeks<br>3 group sessions/wk led by qigong expert<br>Participants received DVD to practice at home 1-1.5hrs/day on days group sessions did not meet	Between-group differences in favor of intervention in Weight (p<0.01);<br>Effect of qi-gong on improved IR was mediated by reduced weight<br>*Combination of qigong expert-led group practice and at-home DVD-led individual practice.*<br>*Qigong practice fluctuated throughout the study.*
**SUN et al. (2010)**<br>**USA/SEATTLE, WA**<br>*Friere 2013*	Yi Ren Medical Qigong (YRMQ) defines qigong as traditional Chinese energy medicine practice combining breathing, movement, and meditation. "The term 'qi' (or 'chi') meaning 'vital energy of the body' and 'gong' meaning the skill and achievement cultivated through regular and disciplined practice."	12 weeks<br>Weekly Qigong or PRT group sessions for 60min/wk with certified instructors and practiced at least twice a week at home for 30min following a DVD	All qigong intervention participants showed reduction in FBG in contrast to the control and PRT groups; YRMQ group showed significant reductions in FBG, slight reduction in weight and a trend toward reversing insulin resistance.<br>*Specific exercise descriptions lacking from publications making it difficult to replicate study.*<br>*Combination of Certified Qigong Instructor-led group practice and at-home DVD-led individual practice.*
**TSUJIUCHI et al. (2002)**<br>**JAPAN/TOKYO**<br>*Xin 2007*<br>*Lee 2009*<br>*Friere 2013*	Qi-Gong relaxing consists of "controlled synchronized breathing with slow body movements as an aerobic exercise and relaxation"	4 months<br>Weekly 2hour Qi-gong group sessions held by a Chinese Qi-gong doctor; all requested to practice at home	Compared with the Control period of Group 2, Group 1 demonstrated significant improvement of HbA1c level (p< 0.01)<br>*Combination of Chinese Qi-gong doctor-led group practice and at-home individual practice*
**XIAO (2017)**<br>**CHINA/BEJING**	Ba duan jin Qigong (BDJ) -<br>60min training –<br>10min warming-up<br>BDJ exercise 45min<br>5min cool-down	24 weeks<br>5-1-2hr sessions/wk	HbA1c (p=0.034) at baseline and week24<br>*Little known about medications, diet and additional exercise in subjects.*<br>*Published photos to demonstrate qigong exercises along with step-by-step procedures.*
**YOUNGWANICHSETHA et al. (2013)**<br>**SOUTHERN THAILAND**	Tai Chi Qigong Group protocol:<br>1. warm-up with 25 movements<br>2. tai chi qigong following set one of Lin Housheng's style comprising 18 movements for 30min<br>3. cooling down with 5 qigong movements for 5min	12 weeks<br>Intervention Group:<br>50min tai chi qigong exercise program 3x/wk for 12weeks plus 5x/wk at home - 3-6months postpartum<br>Control Group:<br>usual care	Statistically significant reduction in FPG, HbA1c and BP in intervention when compared with the Control Group (p<0.05). No significant between-group differences in body-weight or BMI at 12weeks.<br>*Subjects all post-partum younger women making study not generalizable, but helpful for this population.*<br>*Combination of group and individual practice.*
